# Transcriptomic profiling reveals distinct molecular signatures among lesion types in hidradenitis suppurativa

**DOI:** 10.3389/fimmu.2025.1715474

**Published:** 2026-02-13

**Authors:** Hakim Ben Abdallah, Christine Daugaard, Louise Schøsler, Mads Kirchheiner Rasmussen, Line Kibsgaard, Trine Høgsberg, Claus Johansen, Trine Bertelsen

**Affiliations:** Department of Dermatology and Venereology, Aarhus University Hospital, Aarhus, Denmark

**Keywords:** hidradenitis suppurativa, acne inversa, transcriptomics, lesion heterogeneity, inflammatory pathways, precision medicine

## Abstract

**Introduction:**

Hidradenitis suppurativa is a painful inflammatory disease characterised by diverse cutaneous lesions that exhibit distinct clinical morphologies. The study aimed to examine the transcriptome of the various hidradenitis suppurativa lesion types.

**Methods:**

Transcriptomic analyses were performed using bulk RNA sequencing to examine the various lesion types, including draining tunnels, inflammatory nodules, non-draining tunnels, non-lesional skin from patients with hidradenitis suppurativa and normal skin from healthy individuals.

**Results/discussion:**

Principal component and differential expression analyses revealed that these different lesion types exhibit distinct expression profiles while sharing common transcriptional features. Enrichment analysis of exclusively differentially expressed genes for each lesion type showed increased antigen presentation and cytotoxic T cell response in inflammatory nodules, including genes such as *CD8B*, *HLA-C*, and *HLA-F.* In accordance, cell-type enrichment analysis showed an elevated CD8^+^ T cell signature in inflammatory nodules. Gene set variation analysis demonstrated distinct inflammatory pathway signatures in hidradenitis suppurativa lesions, including heightened IL-1 signalling in draining tunnels with significant upregulation of *IL1B* and its receptor *IL1R*, but not *IL1A.* Furthermore*, IL17A* and *IL17F* were significantly increased in draining tunnels, inflammatory nodules, and non-draining tunnels, with *IL17F* showing the highest expression in draining tunnels. *MMP3*, *MMP8*, and *MMP10* were preferentially upregulated in draining tunnels, indicating a role in tunnel activity. Moreover, *AQP4* and *AQP5* were significantly downregulated in both lesional and non-lesional hidradenitis suppurativa skin, suggesting a possible involvement in preclinical pathogenic events.

**Conclusion:**

Distinct molecular signatures specific for each lesion type were identified, which may reflect differences in molecular mechanisms. These findings underscore the importance of distinguishing between lesion types in translational and clinical studies.

## Introduction

Hidradenitis suppurativa (HS) is a chronic, relapsing inflammatory disease that usually affects intertriginous body sites such as the axilla and groin (1). It is characterised by heterogeneous lesion types including inflammatory nodules and abscesses that may progress to the formation of tunnels and excessive scarring (2). Despite the relatively high prevalence (estimated at approximately 1%) and substantial impact on quality of life, the pathophysiology of hidradenitis suppurativa remains inadequately understood, impeding the development of effective therapeutic strategies (3, 4).

To date, biologic therapies targeting TNF or IL-17 have been approved for the treatment of hidradenitis suppurativa; however, only a relatively modest percentage of patients achieve near-complete remission of skin symptoms (5–7). Given the limited efficacy of existing therapies, there is a pressing need to further elucidate the pathogenic molecular mechanisms underlying HS to uncover novel therapeutic strategies.

Recent transcriptomic studies have started to unravel the complex molecular landscape of HS, revealing widespread dysregulation of genes and pathways (8–15). These studies have highlighted broad activation of the innate and adaptive immune system with T cells and B cells (13, 15), elevated expression of cytokines (e.g., *IL1B*, *IL17A*, *IL17F*) (9, 13, 16), antimicrobial peptides (e.g., *DEFB4A*) (10, 12, 13), matrix metalloproteinases (eg., *MMP1*, *MMP3*) (11) and downregulation of genes associated with apocrine sweat gland function (eg., *AQP5*) (10, 12).

However, the heterogeneous HS lesion types exhibit distinct clinical morphologies (2), which may reflect differences in molecular profile and underlying pathomechanisms. To investigate this, we performed a transcriptomic analysis using bulk RNA sequencing to examine the molecular profiles among various HS lesion types (draining tunnels, inflammatory nodules, non-draining tunnels), non-lesional HS skin and normal skin from healthy individuals.

## Materials and methods

### Biopsies

Three-millimetre punch biopsies were collected from draining tunnels (purulent discharge at rest or to palpation) and non-draining tunnels (no purulent discharge) from 20 HS patients and normal skin from eight healthy donors. Additionally, biopsies previously collected from inflammatory nodules (nodules > 1 cm, tender to palpation and erythematous) and non-lesional skin (uninvolved skin at least 10 cm from the active lesions) from 15 other HS patients were jointly analysed (15). All biopsies underwent an identical methodological workflow. The paired biopsies were obtained from the same inguinal or axillary regions and immediately snap-frozen in liquid nitrogen. The patients were recruited from the Department of Dermatology and Venereology, Aarhus University Hospital in Denmark. Written informed consent was obtained from the patients. The Central Denmark Regional Ethical Committee approved the study protocol (no. 1-10-72-107-24).

### RNA isolation

Upon RNA purification, the snap-frozen biopsies were placed in RNAlater-ICE (ThermoFisher) at -20 °C overnight. The biopsies were added to SV RNA lysis buffer with β-mercaptoethanol (SV Total RNA Isolation System; Promega, Madison, WI, USA) and homogenised using TissueLyser (Qiagen, Hilden, Germany). The remaining RNA purification steps continued as per the manufacturer’s instructions. RNA quality and quantity were determined with Bioanalyzer 2100 (Agilent Technologies, Santa Clara, CA, USA) and Nanodrop 2000 (ThermoFisher), respectively.

### Bulk RNA sequencing

Eurofins Genomics Europe Sequencing GmbH (Konstanz, Germany) performed paired-end RNA sequencing of total RNA according to their protocols. NEBNext Ultra II Directional RNA Library Prep Kit with 100 ng total RNA was used to prepare the RNA library. Illumina NovaSeq 6000 platform with 2x150 Sequence mode was used to sequence at least 20 million read pairs. FastQC (version 0.12.0) was used for the quality control of raw sequence data. R package Subread (version 2.10.3) was used to align raw reads to the human reference genome (GRCh38.p13 primary assembly) (17). Gene counts were determined by uniquely aligned unambiguous reads. Genes with more than five samples with normalised counts greater than or equal to 5 were included in the dataset for further analysis. Variance stabilised transformed data (vst) performed by the R package DESeq2 (1.36.0) was used for downstream analyses such as principal component analysis (PCA) (18). Differential expression analysis was performed with the Wald test and independent filtering enabled in DESeq2 (18). False discovery rate (FDR)-adjusted *P*-values < 0.05 and |log2(fold change)| > 1 were the criteria for differentially expressed genes (DEGs).

Over-representation analysis (ORA) with Gene Ontology biological processes (GO: BP) and KEGG gene sets was performed using DAVID and validated with ShinyGO 0.80 (19, 20). Gene set enrichment analysis (GSEA) was performed with all genes from the differential expression analysis ranked by log_2_(fold change)*-log_10_(p-value) using fsgea (version 1.28.0) (21). Gene set variation analyses (GSVA) were performed with the R package GSVA (version 1.46.0) (22). Reactome and GO: BP gene sets were imported from The Molecular Signatures Database (MSigDB) through msigbr (version 7.5.1) (22), whereas gene sets associated with T helper cells (Th1, Th2, Th17, Th22) were based on previously published studies (23–26). Cell types enrichment analysis was conducted using xCell webtool on TPM (transcripts per million kilobase) normalized dataset (27).

### Statistics

For single-group comparisons comparing HS lesions and non-lesional skin with normal skin, One-way ANOVA with Dunnett’s *post-hoc* test was performed. P-values < 0.05 were considered significant. All statistical analyses were performed with GraphPad Prism 10.0.1 and R 4.2.0 software.

## Results

**Figure 1:**
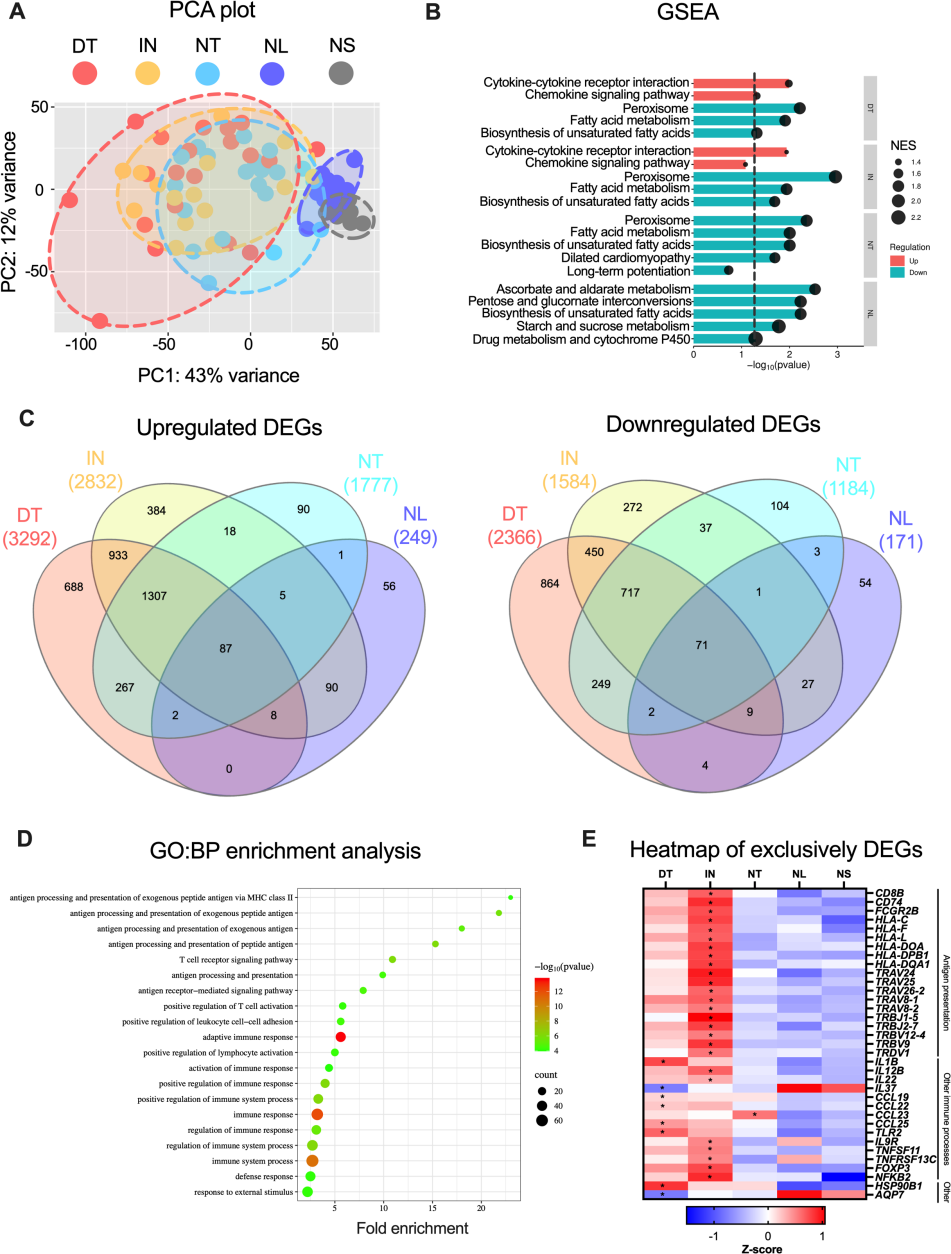
Bulk RNA sequencing analysis of draining tunnels (DT; *n* = 20), inflammatory nodules (IN; *n* = 15), non-draining tunnels (NT; *n* = 20), non-lesional skin (NL; *n* = 15) from hidradenitis suppurativa patients and normal skin from healthy individuals (NS; *n* = 8). **(A)** Principal component analysis using variance stabilising transformed (vst) data. **(B)** Gene set enrichment analysis showing the top 5 significant KEGG pathways for the HS lesions and NL compared with NS. The dashed vertical line depicts a FDR-adjusted p-value of 0.05. The size of the dots represents the normalised enrichment scores (NES), whereas the colour of the graphs indicates positive (teal) or negative (red) NES values. **(C)** Venn diagrams of upregulated (left panel) and down-regulated (right panel) differentially expressed genes (DEGs; |log_2_[fold change]| > 1 and FDR-adjusted p-value < 0.05) compared with NS. **(D)** Overrepresentation analysis (ORA) of upregulated DEGs in IN compared with NS, showing the top 20 significant Gene Ontology biological processes (GO: BP) terms. **(E)** Heatmap displaying z-scores of gene expression for a subset of exclusively DEGs. Asterisks (*) denote DEGs. DEGs, differentially expressed genes; DT, draining tunnels; IN, inflammatory nodules; NES, normalised enrichment factor; NL, non-lesional skin; NS, normal skin; NT, non-draining tunnels. ORA.

To investigate the transcriptomic differences, an integrative analysis was performed with bulk RNA sequencing data from skin biopsies of draining tunnels (*n* = 20), non-draining tunnels (*n* = 20) and normal skin (*n* = 8). These samples were jointly analysed with inflammatory nodules (*n* = 15) and non-lesional skin biopsies (*n* = 15) from a prior study that followed the exact same experimental workflow (15). Compared to normal skin, the differential expression analysis identified, respectively, up- and downregulated DEGs corresponding to 3292 and 2366 DEGs in draining tunnels, 2832 and 1584 DEGs in inflammatory nodules, 1777 and 1184 DEGs in non-draining tunnels, and 249 and 171 DEGs in non-lesional skin (**Supplementary Table S1**). Principal component analysis (PCA) showed distinct neighbouring clustering of non-lesional and normal skin. The draining tunnels, inflammatory nodules and non-draining tunnels showed trends of clustering with marked overlap, indicating that these lesion types exhibit distinct expression profiles while sharing common transcriptional features (**Figure 1A**). GSEA revealed that the top 5 most enriched KEGG pathways were related to upregulation of inflammation in draining tunnels and inflammatory nodules (*Cytokine-cytokine receptor interaction*, *chemokine signalling pathway)* and downregulation of metabolic processes, in particular lipid metabolism (*fatty acid metabolism*, *biosynthesis of unsaturated fatty acids*), in all HS lesions compared with normal skin (**Figure 1B**). In accordance with the PCA plot, the Venn diagrams illustrate that while the majority of DEGs are mutually expressed, a notable number of up- and downregulated DEGs were exclusively expressed, respectively, 688 and 864 DEGs in draining tunnels, 384 and 272 DEGs in inflammatory nodules, 90 and 104 DEGs in non-draining tunnels, and 56 and 54 DEGs in non-lesional skin (**Figure 1C**, **Supplementary Table S2**). To complement these analyses versus normal skin, direct lesion comparisons (DT vs NT, DTvs IN, and IN vs NT) are available in **Supplementary Table S3**.

To examine the functions of the exclusively DEGs, overrepresentation analysis (ORA) with GeneOntology was performed (**Supplementary Table S4**). Significantly enriched pathways were identified for upregulated exclusively DEGs in inflammatory nodules, unlike the other HS lesions. The top 20 significant terms were all related to inflammation, especially for antigen presentation and T-cell activity (**Figure 1D**).

In draining tunnels, upregulated exclusively DEGs included *IL1B*, chemokines attracting macrophages (*CCL25*, *CCL19*, *CCL22*), Toll-like receptor 2 (*TLR2*), the endoplasmic reticulum-associated heat-shock protein 90 GRP94 (*HSP90B1*), and downregulation of the anti-inflammatory interleukin *IL37* belonging to the IL-1 superfamily and aquaporin *AQP7* (**Figure 1E**). In inflammatory nodules, upregulated exclusively DEGs included pro-inflammatory interleukins (*IL22*, *IL12B*), the CD8 co-receptor (*CD8B*) involved in cytotoxic T cell antigen presentation with MHC class I molecules, the invariant chain (*CD74*) serving as chaperone necessary for MHC II antigen presentation, several HLA genes encoding proteins for MHC I (e.g., *HLA-C*, *HLA-F*) and MHC II (*HLA-DOA*, *HLA-DRB1*), and T cell receptor variable chains (e.g., *TRAV24*, *TRBJ1*-5, *TRDV1*, *TRGV4*). In non-draining tunnels, *CCL23* was exclusively upregulated, a chemokine attracting T cells, monocytes, neutrophils and a mediator of angiogenesis.

Taken together, these findings indicate that the different HS lesion types display distinct transcriptomic profiles with several key genes exclusively expressed, and antigen presentation with T cell activation appears to be more prominent in inflammatory nodules.

**Figure 2:**
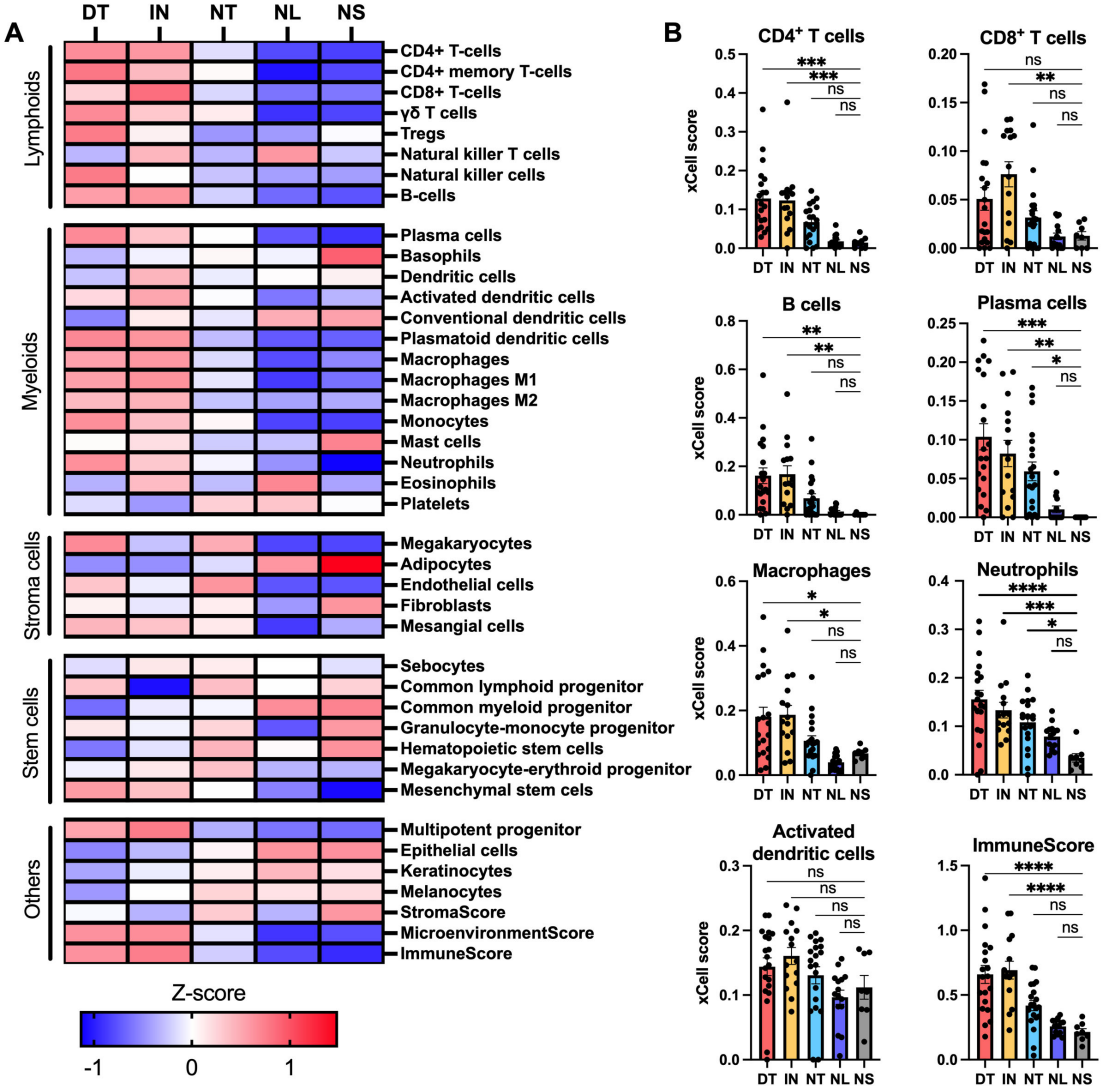
Cell type enrichment analysis of bulk RNA sequencing data using xCell. **(A)** Heatmap showing xCell enrichment z-scores for 38 relevant cell types. **(B)** Bar graphs showing xCell scores for CD4^+^ T cells, CD8^+^ T cells, B cells, plasma cells, macrophages, neutrophils, activated dendritic cells and the ImmuneScore. Composite scores were calculated for immune cells (ImmuneScore), stromal cells (StromaScore), and the combined contribution of immune and stromal cells (MicroenvironmentScore) using xCell. Data are presented as mean ± SEM. * p < 0.05 **p ≤ 0.01, ***p ≤ 0.001, ****p ≤ 0.0001. ns, not significant. DT, draining tunnels; IN, inflammatory nodules; NL, non-lesional skin; NS, normal skin; NT, non-draining tunnels.

The abundance of major cell types was estimated by calculating the cell type signature enrichment scores using xCell (**Figure 2A**). Several key inflammatory cell types were significantly enriched in draining tunnels and inflammatory nodules compared with normal skin, including CD4^+^ T cells, B cells, plasma cells, macrophages and neutrophils, along with the ImmuneScore (a composite score for immune cells) (**Figure 2B**). However, the cell enrichment for CD8^+^ cells was only significantly increased in inflammatory nodules, further supporting that cytotoxic T cell response may be more pronounced in the early stages of skin inflammation in HS. Notably, plasma cells and neutrophils were significantly enriched in non-draining tunnels, illustrating increased immune cell infiltration despite no clinically visible active inflammation.

**Figure 3:**
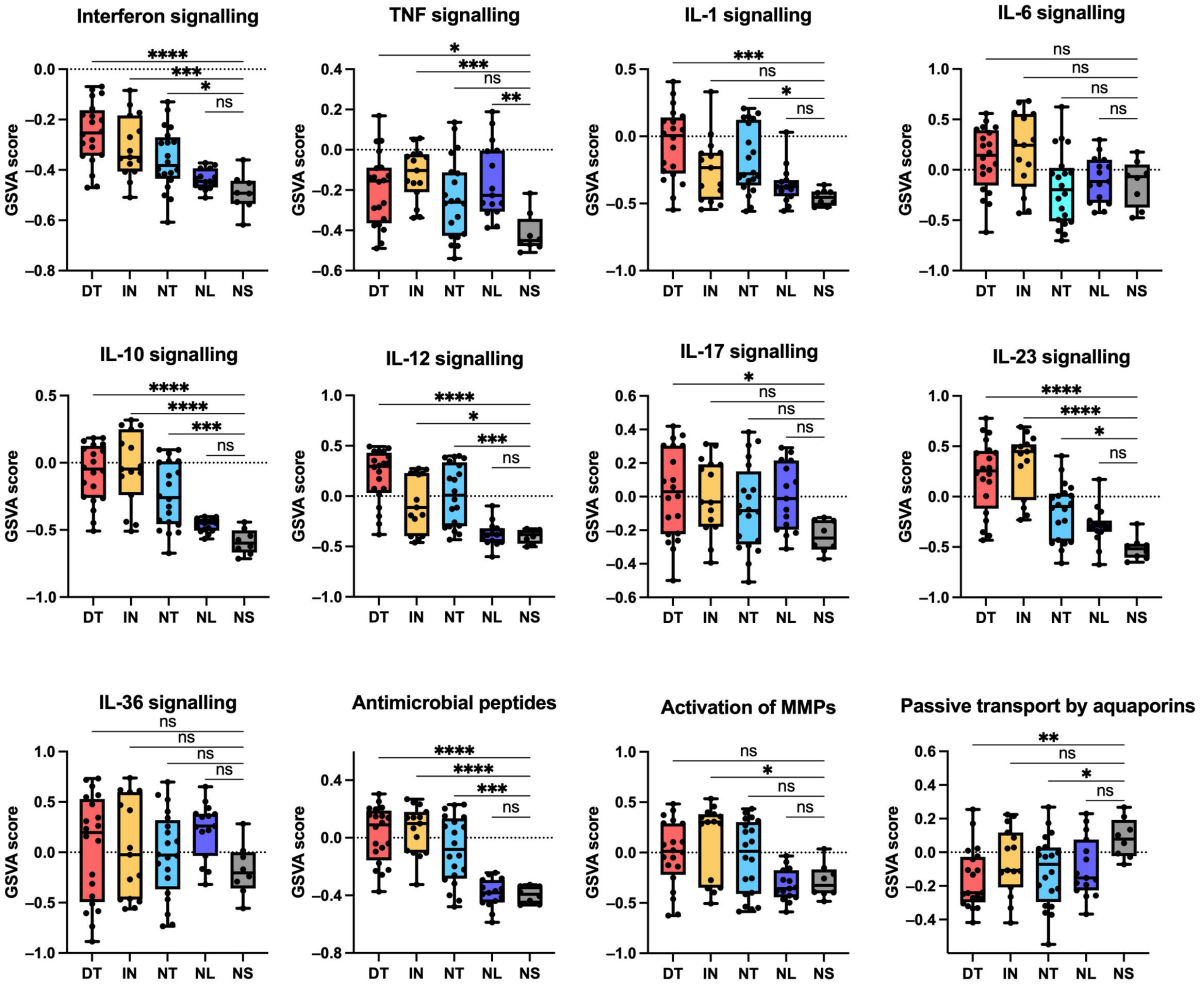
Gene set variation analysis (GSVA) of key Reactome pathways based on bulk RNA sequencing data. Data are shown as a boxplot with whiskers ranging from the minimum to the maximum data point. * p < 0.05 **p ≤ 0.01, ***p ≤ 0.001, ****p ≤ 0.0001; ns, not significant. Note: GSVA scores should be interpreted relative to lesion-type differences rather than as absolute values. DT, draining tunnels; IN, inflammatory nodules; NL, non-lesional skin. NS, normal skin; NT, non-draining tunnels.

**Figure 4:**
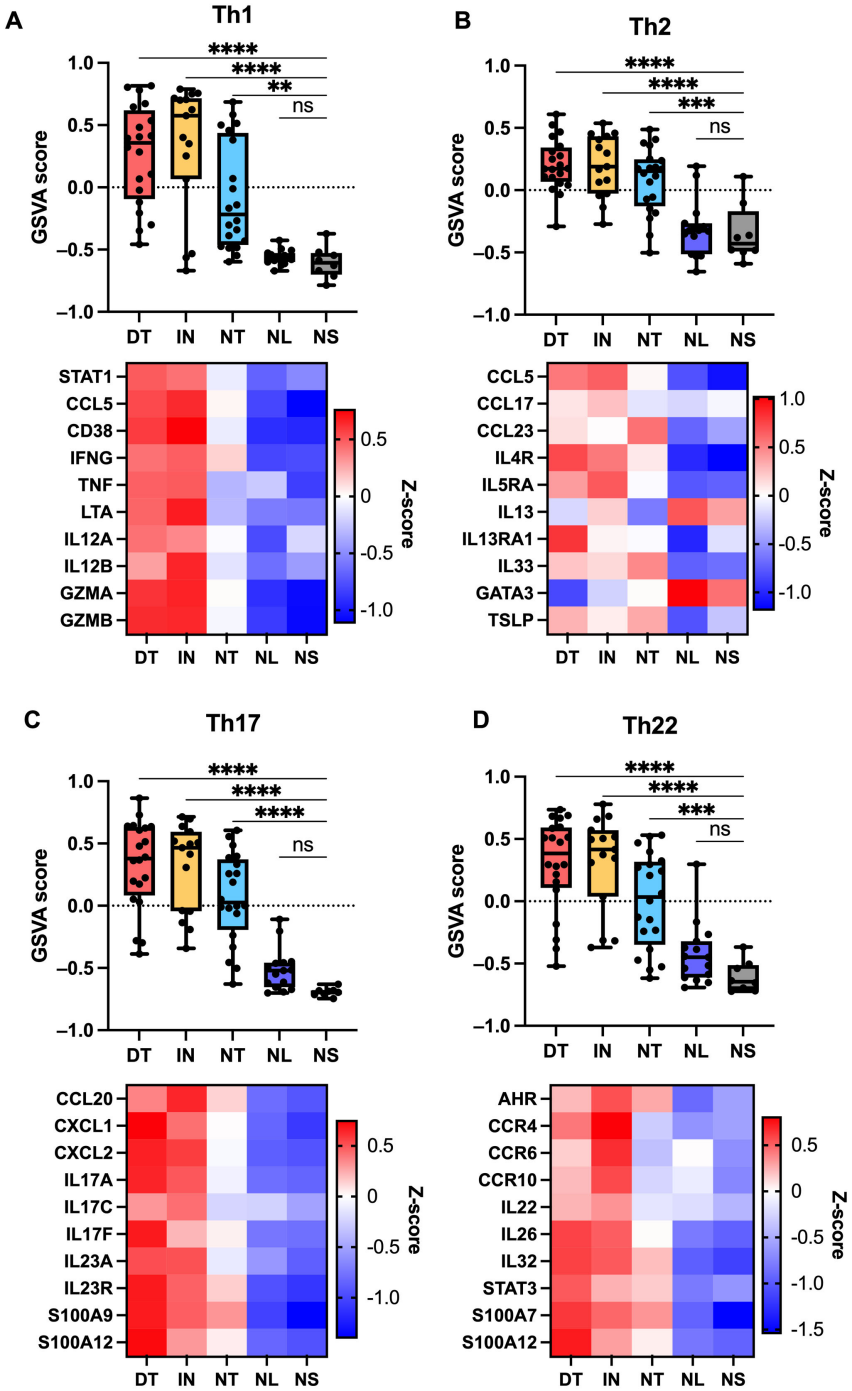
Th cell signature analysis based on bulk RNA sequencing data using gene set variation analysis (GSVA) and custom gene sets related to **(A)** Th1-, **(B)** Th2-, **(C)** Th17- and **(D)** Th22 responses. GSVA scores are shown in the upper panels, while heatmaps illustrating expression levels of genes within the corresponding gene sets are shown in the lower panels. **p ≤ 0.01, ***p ≤ 0.001, ****p ≤ 0.0001; ns, not significant. GSVA scores should be interpreted relative to lesion-type differences rather than as absolute values. DT, draining tunnels; IN, inflammatory nodules; NL, non-lesional skin; NS, normal skin; NT, non-draining tunnels.

To explore the activity of key pathways, a gene set variation analysis (GSVA) was performed using Reactome pathways (**Figure 3**). Multiple pathways were significantly enriched in draining tunnels and inflammatorynodules, including interferon, TNF, IL-10, IL-12 and IL-23 pathway signalling. In accordance, gene expression of *TNF*, *IFGN*, *IL10* and *IL23* were significantly increased; however, *IL12B* gene expression was only significantly increased in inflammatory nodules (**Supplementary Figure S1**). The IL-1 signalling pathway was significantly enriched in draining tunnels andnon-draining tunnels. *IL1B* and the receptor *IL1R* were significantly increased in draining tunnels, whereas no changes were detected for *IL1A* expression. Although IL-6 pathway signalling was not significantly enriched, *IL6* gene expression was elevated in HS lesions. The IL-17 signalling pathway was significantly elevated in draining tunnels and non-significantly increased in the other HS lesion types. Further examination revealed that *IL17A* and *IL17F* were significantly upregulated in draining tunnels, inflammatory nodules and non-draining tunnels, with the highest expression of *IL17F* observed in draining tunnels (**Supplementary Figure S1**). *IL17C* was slightly increased but did not reach statistical significance. In contrast, *IL17D* was downregulated, while no changes were detected for *IL17B* and *IL17E*. Among IL-17 receptor subunits, *IL17RA* was upregulated in inflammatory nodules, whereas *IL17RB*, *IL17RC*, *IL17RD* and *IL17RE* were downregulated across the HS lesions. MMPs demonstrated marked differences in gene expression. *MMP1*, *MMP9*, *MMP11*, *MM12*, *MM13*, *MM14*, *MMP15*, *MMP17*, *MMP19*, *MMP25* were significantly increased in draining tunnels and inflammatory nodules, whereas *MMP3*, *MMP8*, *MMP10* were exclusively upregulated and *MMP27* and *MMP28* were exclusively downregulated in draining tunnels. The Reactome gene set for passive transport by aquaporins was significantly downregulated in draining tunnels and non-draining tunnels. The changes in gene expression revealed that *AQP4*, *AQP5* and *AQP6* were significantly downregulated in draining tunnels, inflammatory nodules and non-draining tunnels, whereas *AQP7* and *AQP9* were exclusively downregulated in draining tunnels. *AQP4* and *AQP5* were also downregulated in non-lesional skin, which may indicate a role in the early pathogenesis of HS. Furthermore, GSVA analysis of Th signatures demonstrated significant upregulation of Th1/2/17/22 signature in draining tunnels, inflammatory nodules and non-draining tunnels compared with normal skin (**Figure 4**). While significantly upregulated, the GSVA score difference was modest for Th2, suggesting that the Th1/17/22 pathways are more predominant in HS lesions. Moreover, the Th1 pathway appeared to be most prominent in inflammatory nodules, which is important for the activation of CD8^+^ T cells. These findings suggest that widespread immune activation is present in HS lesions.

In summary, the different HS lesion types displayed distinct inflammatory pathway signatures, such as increased IL-1 signalling in draining tunnels, and marked differences in the expression of MMPs and aquaporins, which may reflect unexplored pathogenetic mechanisms.

## Discussion

Our comprehensive molecular profiling of different HS lesion types discovered a widespread immune dysregulation with distinct molecular features for each lesion type. While draining tunnels and inflammatory nodules exhibited the strongest immune activation, notable molecular alterations were also observed in non-draining tunnels and non-lesional skin, indicating that significant molecular changes can be present in clinically non-inflamed skin. These findings highlight the molecular heterogeneity underlying the HS lesion types and underscore the importance of distinguishing between specific lesion types when performing translational and clinical studies. Such stratification may lead to more consistent results and novel insights into the complex pathogenesis.

An increased cytotoxic T cell response was observed in inflammatory nodules, as supported by the exclusively DEGs (e.g *CD8B*, *HLA-C*, *HLA-F*) and the increased cell type enrichment for CD8^+^ T cells. Other studies have shown increased CD8^+^ infiltration with production of TNF and IFN-γ in active HS lesional skin (14, 28). Furthermore, immunohistology investigations have identified subepidermal and perifollicular CD8^+^ T cell infiltration in very early HS lesions (29). These findings may indicate that CD8^+^ T cell activation may be implicated in the early pathogenesis of HS. While the mechanism of activation remains unclear, microbial antigens and autoantigens from stressed cells could be cross-presented by antigen-presenting cells on MHC class I molecules to activate CD8^+^ T cells. Recently, increased levels of autoantibodies have been detected, suggesting autoreactivity in HS (30, 31). Natural killer T cells (NTK) and natural killer cells (NK) are other cells with cytotoxic properties that have recently been proposed to be major drivers of HS disease pathogenesis, including tunnel formation and fibrosis (32, 33). Interestingly, our cell type enrichment analysis identified a non-significant increase of NKT in inflammatory nodules and non-lesional skin, indicating a possible role in early HS lesions, whereas NK was significantly increased in draining tunnels, supporting previous findings with a possible role in tunnel formation (33).

The IL-1 family cytokines have been shown to be involved in HS, contributing to the inflammatory response by promoting immune cell infiltration and secretion of MMPs, which may facilitate rupture of dilated hair follicles, abscess and tunnel formation (34). Macrophages stimulated through TLRs, such as TLR2, have been shown to be significant producers of IL-1β (35, 36). Consistent with our findings, a recent transcriptome meta-analysis of HS showed that *TLR2*, *IL1B*, *IL36A* and *IL36G* were among the upregulated DEGs, whereas *IL37* was downregulated (11). Our GSVA analysis revealed that the IL-1 signalling pathway was significantly increased in draining tunnels and non-draining tunnels. Furthermore, *IL1B*, the receptor *IL1R1* and *TLR2* were exclusively significantly upregulated and the anti-inflammatory *IL37* was exclusively downregulated in draining tunnels. These findings suggest that IL-1 signalling is more predominant in draining tunnels, which may correspond to a better therapeutic response to IL-1 family antagonism in draining tunnels. Several clinical studies have evaluated IL-1 family antagonism with variable success (37, 38). In a phase II randomised controlled trial (RCT), treatment with anakinra (an IL-1 receptor antagonist) resulted in 78% (7 out of 9) of participants achieving HiSCR 50 compared with 30% (3 out of 10) in the placebo group (p = 0.04), but the reduction in lesion types was not specified (39). IL-36 receptor antagonists (Imsidolimab and Spesolimab) have failed to demonstrate significant improvement in AN count (inflammatory nodules and abscess) and HiSCR50 in phase 2 II trials; however, Spesolimab led to a notable reduction in draining tunnels (-40.1%) in contrast with an increase in the placebo arm (56.6%) (40, 41). Lutikizumab (Anti IL-1 α/β) and Spesolimab are currently in phase II (NCT05139602) and II/III trials (NCT05819398), respectively, which may further show a possible better therapeutic response in draining tunnels compared with other HS lesions.

Consistent with previous findings (11), we observed significant dysregulation of several MMPs, particularly in draining tunnels and inflammatory nodules. These findings support that MMPs contribute to both the acute inflammatory microenvironment of inflammatory nodules and the chronic tissue destruction and remodelling in tunnels. Notably, *MMP3, MMP8*, and *MMP10* were preferentially upregulated in draining tunnels, suggesting a potential role in the development or maintenance of tunnel formation. MMP8 (also known as neutrophil collagenase) is produced by both neutrophils and fibroblasts, and its blood levels have been shown to correlate with HS disease severity (42). The distinct expression patterns of specific MMPs suggest differential roles in HS pathogenesis; however, further studies are needed to clarify their functional contributions.

AQPs are implicated in fluid homeostasis, epithelial barrier function and glandular secretion and recently understood to be involved in cellular processes and intracellular signalling pathways (43). AQPs have been found to be downregulated in HS (11, 12, 44). Interestingly, we observed that *AQP4* and *APQ5* were significantly downregulated in both HS lesions and non-lesional skin, indicating that these two AQPs may play a role in the early pathogenic events preceding the clinical manifestations. AQP5 are expressed in sweat glands, and impaired sweat gland function has been proposed to contribute to the HS pathogenesis (11, 12, 45). Other studies have shown that AQP5 expression in keratinocytes (HaCAT) regulates proliferation and differentiation, indicating functions beyond sweat gland function (46). AQP4 is predominantly studied in the CNS, but it is also expressed throughout epithelial tissues in the body; however, the role in the skin remains unknown (47, 48).

Our findings demonstrate increased Th response in all HS lesions, though the Th1/17/22, pathways were predominant, suggesting broad immune activation, even in non-draining tunnels. While HS has been associated with a predominantly Th1/17 response (49), we also identified a strong Th22 response, however the current literature on the role of IL-22 in HS is conflicting (50). Several studies have reported increased *IL22* expression and Th22 response in the skin (9, 51, 52), but decreased serum levels of IL-22 have also been reported in HS patients (53).

*IL13* was not upregulated in HS lesions, and *IL4* expression was below the filtering threshold and therefore not included in the dataset. The Th2 enrichment was instead driven by other genes such as *TSLP*, *CCL5*, *CCL17* and *IL33*. This likely reflects generalised immune activation and epithelial cell damage rather than a classical Th2-driven response.

Limitations to this study include that analyses on protein levels were not performed; however, mRNA expression and protein levels have been shown to be significantly correlated in HS (54). HS lesions were identified based on established clinical definitions; however histological confirmation would further strengthen the accuracy of lesion classification. A key limitation of this study is the absence of functional validation. While we identify distinct transcriptional patterns across lesion types, further studies are needed to assess the mechanistic and clinical significance. Moreover, clinical information such as gender, age, smoking status, ethnicity and clinical phenotypes was not taken into consideration in the analyses.

In conclusion, our comprehensive molecular profiling of distinct HS lesion types revealed widespread immune dysregulation across all lesions with unique molecular signatures specific to each lesion type. Notably, IL-1 signalling was predominant in draining tunnels, while antigen presentation and cytotoxic T cell responses were more prominent in inflammatory nodules. These findings highlight the molecular heterogeneity of HS lesion types. Further functional studies are warranted to explore whether the lesion types may be driven by distinct molecular mechanisms with potential clinical and therapeutic implications.

## Data Availability

The datasets presented in this study can be found in online repositories. The names of the repository/repositories and accession number(s) can be found below: https://www.ncbi.nlm.nih.gov/geo/, (GSE315442 and GSE249027).

## Supplementary material

Supplementary Figure 1 RNA sequencing results showing log2(normalised counts) of relevant genes. Data are presented as mean + SEM. Statistical significance was assessed using one-way ANOVA followed by Dunnett’s *post-hoc* test, with normal skin (NS) as the control group. * p < 0.05 **p ≤ 0.01, ***p ≤ 0.001, ****p ≤ 0.0001; ns, not significant. DT, draining tunnels. IN, inflammatory nodules. NL, non-lesional skin. NS, normal skin. NT, non-draining tunnels.

Supplementary Table 1 Differentially expressed genes in draining tunnels, inflammatory nodules, non-draining tunnels and non-lesional hidradenitis suppurativa skin, each compared with normal skin from healthy individuals.

Supplementary Table 2 Exclusively differentially expressed genes unique in draining tunnels, inflammatory nodules, non-draining tunnels and non-lesional hidradenitis suppurativa skin, each compared with normal skin from healthy individuals.

Supplementary Table 3 Differential gene expressions analyses comparing draining tunnels vs non-draining tunnels, draining tunnels vs inflammatory nodules, and inflammatory nodules vs non-draining tunnels.

Supplementary Table 4 Results of overrepresentation analysis performed on the exclusively differentially expressed genes from draining tunnels, inflammatory nodules, non-draining tunnels, and non-lesional HS skin compared with normal skin from healthy individuals.
