# Supplementary figures and images for: Transcriptomic profiling reveals distinct molecular signatures among lesion types in hidradenitis suppurativa
Source: Front Immunol. 2026 Feb 13;16:1715474. doi: 10.3389/fimmu.2025.1715474 (PMC12946153; doi:10.3389/fimmu.2025.1715474)

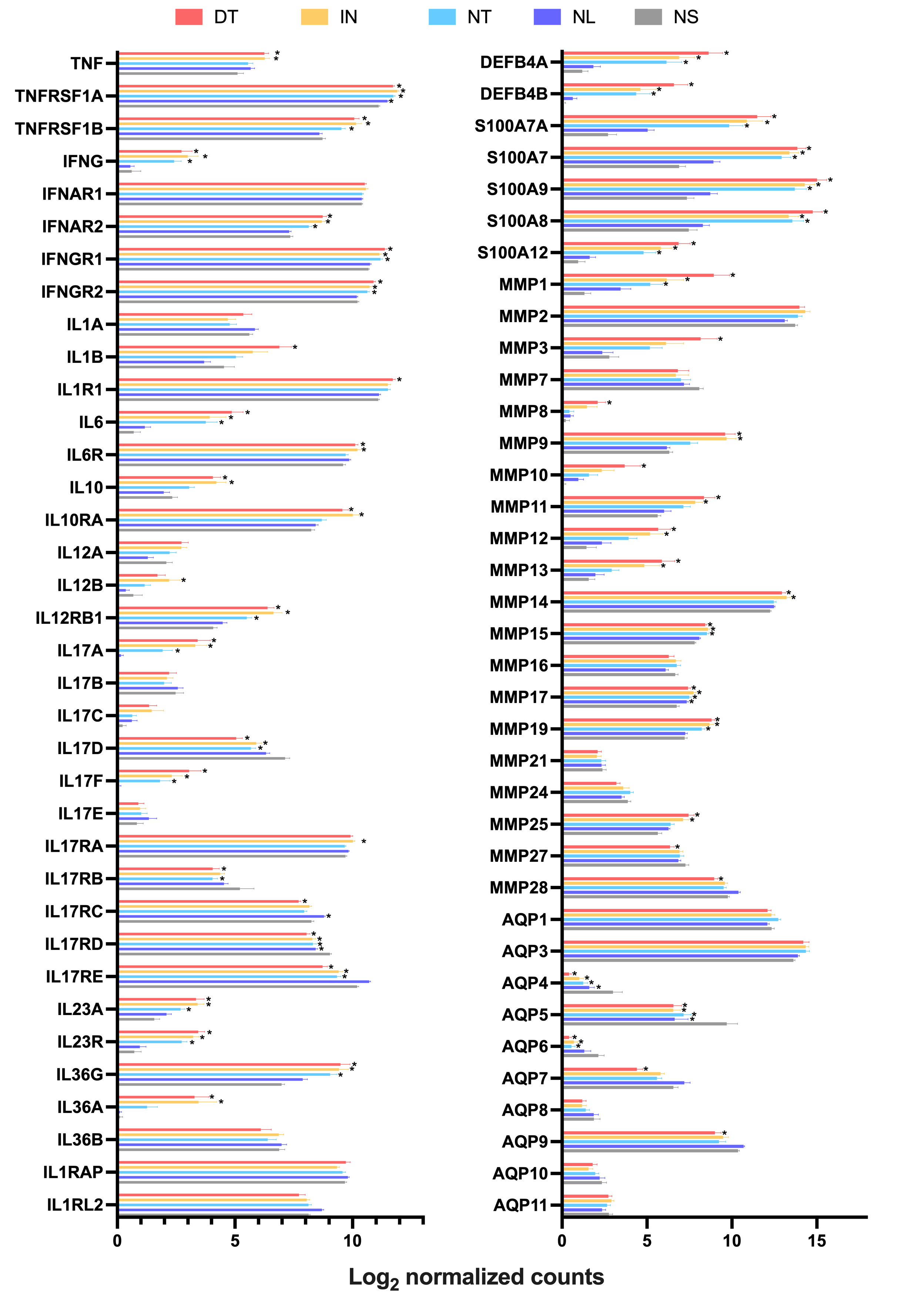

Supplement: Supplementary Figure 1 — RNA sequencing results showing log2(normalised counts) of relevant genes. Data are presented as mean + SEM. Statistical significance was assessed using one-way ANOVA followed by Dunnett’s post-hoc test, with normal skin (NS) as the control group. * p < 0.05 **p ≤ 0.01, ***p ≤ 0.001, ****p ≤ 0.0001; ns, not significant. DT, draining tunnels. IN, inflammatory nodules. NL, non-lesional skin. NS, normal skin. NT, non-draining tunnels. [file Image1.tiff]
